# Supplementary material for: A network dynamical simulation model for the study of antibiotic resistance in microbial communities
Source: Gut Microbes. 2026 Sep 19;18(1):2734703. doi: 10.1080/19490976.2026.2734703 (PMC13596919; doi:10.1080/19490976.2026.2734703)
Supplement: Supplementary file 1 [file KGMI_A_2734703_SM9053.zip › supplementary_file_1/Figures/ws_delta_clustering_n100.pdf]

Watts−Strogatz networks: mean change in clustering coefficient (N = 100)

Numbers inside tiles show mean ± SD across simulation replicates

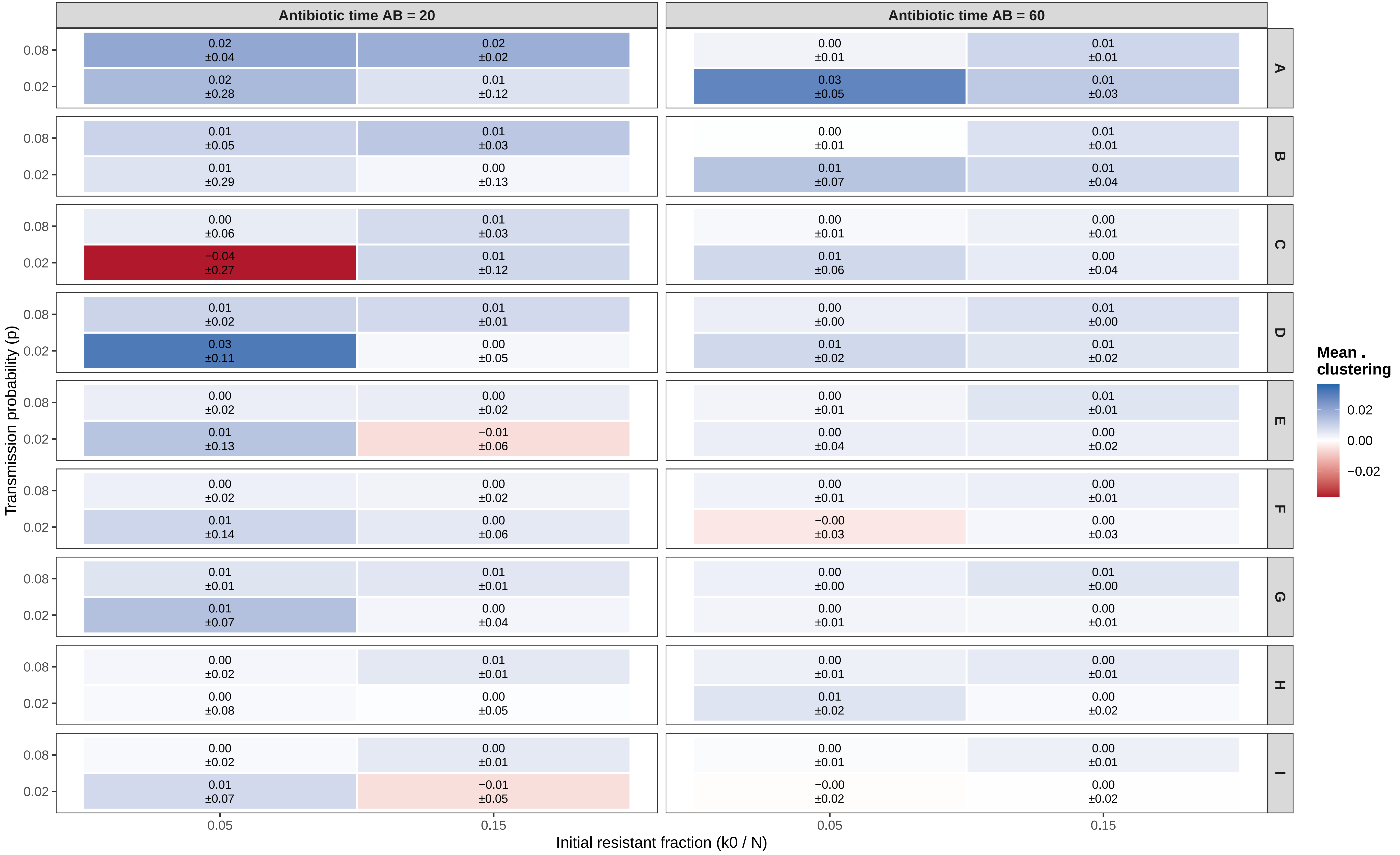

Panel key: A = k 4, beta 0.01 | B = k 4, beta 0.05 | C = k 4, beta 0.1 | D = k 8, beta 0.01 | E = k 8, beta 0.05 | F = k 8, beta 0.1 | G = k 12, beta 0.01 | H = k 12, beta 0.05 | I = k 12, beta 0.1
